# Should We Say Goodbye to Latent Constructs to Overcome Replication Crisis or Should We Take Into Account Epistemological Considerations?

**DOI:** 10.3389/fpsyg.2019.01949

**Published:** 2019-08-27

**Authors:** Barbara Hanfstingl

**Affiliations:** Institute of Instructional and School Development, Alpen-Adria-Universität Klagenfurt, Klagenfurt, Austria

**Keywords:** epistemology, latent constructs, assimilation, accommodation, déjà-variables, assimilation bias, over-accommodation, over-assimilation

## Abstract

This paper discusses theoretical and epistemological problems concerning validity of psychological science in the context of latent constructs. I consider the use of latent constructs as one reason for the replicability crisis. At the moment, there exist different constructs describing the same psychological phenomena side by side, and different psychological phenomena that are reflected by the same latent construct. Hagger called them déjà-variables, which lead to a decreasing validity of measurements and inhibit a deeper understanding of psychological phenomena. To overcome this problem, I suggest a shift of theoretical and epistemological perspective on latent constructs. One main point is the explicit consideration of latent constructs as mental representations, which change objects and are changed by objects via assimilative and accommodative processes. The explicit orientation toward assimilation and accommodation allows the control of normally automatized processes that influence our understanding of psychological phenomena and their corresponding latent constructs. I argue that assimilation and accommodation are part of our research practice anyway and cause the mentioned problems. For example, taking a measurement is an assimilative process, and thus a high measurement error should lead to an increase of accommodative processes. Taking into account these considerations, I suggest consequences for research practices, for individual researchers and for the philosophy of science.

## Introduction

In this paper, I argue that replication problems in empirical psychology are not only due to statistical and methodological artifacts but also due to a lack of epistemological clarity. I structure my argument around the following four points: First, I state validity problems that can emerge when latent constructs are used to explain psychological phenomena when research is oriented mainly toward positivism. Second, I show how these problems can be seen through a different epistemological perspective, namely an adaption of Piaget’s psychogenesis with a focus on assimilation and accommodation. Third, I describe examples from psychological research where the concept of assimilation and accommodation helps to understand phenomena where over-assimilation and over-accommodation disturb the achievement of equilibration. And fourth, I delineate consequences at the level of research methods, researchers and for philosophy of science.

The development, description, and investigation of latent constructs (e.g., personality constructs) is a core focus in psychological research. Despite the high development of statistics, the effective and sustainable validation of latent constructs still remains a huge challenge. The call for a higher validity of latent constructs and their generalizability is an issue that has been discussed over many decades in psychological research and adjacent disciplines **(e.g.,** Sackman, 1974**;** Skinner, 2007**;** Rossiter, 2008**;** Johnston et al., 2014**;** Fiedler, 2017**;** Swami et al., 2017**)**. There are two interrelated reasons why the striving for validity of latent constructs is still one of the main challenges. The first lies in the general tradition of science and scientific practice. According to Bickhard and Campbell (2005), psychology has a strong positivistic tradition which was influenced by Ernst Mach. This does not seem to be a problem at first glance. Burrhus Frederic Skinner, for example, took Mach’s approach “as chief basis for his own positivistic views of science” (Smith, 1986, p. 264). However, a pure positivistic perspective on scientific issues can lead to severe validity problems. As Popper (2005) pointed out the weaknesses of positivism for all scientific disciplines, psychological researchers are affected by this issue in a special way. Bickhard and Campbell (2005) still attribute a high influential power in psychological research to neo-Machian positivism because the idea of operationism fosters a positivistic perspective on psychological issues.

The second reason for the validity problem is the use of latent constructs. Their application to make psychology evidence-based even for non-observable phenomena bears boon and bane at the same time. The boon is that now we can investigate non-observable phenomena empirically. The bane is that when researchers started to focus on statistical procedures to calculate latent constructs, they also started to ignore epistemological rules and knowledge that can be drawn solely by theoretical and logical conclusions. For example, Michell (2013) sees the problem in the focus on constructs and differentiates between psychological scientists and scientists in traditional sciences like physics or chemistry. The first concentrate on constructs while the latter concentrate on theoretical concepts. In other words, a psychological researcher thinks in constructs rather than in theories. However, Sherry (2011) used the example of the development of thermometers, which began with the observation of qualitative temperature observations, to argue that the difference between physics and psychology is not given by the difference between construct and theory. I agree with this argument, with one limitation. There is no doubt that the development process of thermometers and psychometric scales is very similar; the younger the process, the more similarities there seem to be. In the meantime, however, physicists have found theoretical foundations – be it the absolute zero point, Brownian motion or gas laws – which all influence the temperature calculably and thus create a basis for temperature beyond thermometers. In psychology we can at best only inaccurately deduce measurements from theories or theories from measurements.

There is no doubt that methodological rules which dominate construct-based research are mandatory but ultimately insufficient for scientific progress and cannot compensate for epistemological or even theoretical considerations (e.g., Fiedler, 2017). Edelsbrunner and Dablander (2018) could show that psychological modeling and scientific reasoning do not always follow a logical procedure. Heene (2013) describes in a very restrictive way why no approach, neither additive conjoint measurement nor modeling of structural equations or item-response theory, can solve the problem of measurement from a purely mathematical point of view and concludes that perhaps “human cognitive abilities and personality traits are simply not quantitative” (p. 3). Here, I would add the idea that cognitive abilities and personality traits might not solely be quantitative. From a metrological perspective, Uher (2018) shows us which epistemological and methodological aspects in most psychological studies are ignored, with a marked reduction of the validity of those studies as a consequence. Recently, Trendler (2019a) revisited an ongoing debate about the justified use of conjoined measurement in psychological research (see also Krantz and Wallsten, 2019; Michell, 2019; Trendler, 2019b).

To summarize, there is a tendency toward (a) positivism and (b) statistic methodical orientation with a coincident lack of theoretical and epistemological orientation. In this paper, I argue that these two reasons bear one of the main responsibilities for the replication crisis. They account for the problem of many overlapping psychological findings that exist side by side, validated within one methodological approach, but bringing them together on a theoretical level fails plenty of times. In psychological literature, we often find the statement that there is “no single,” “no distinct,” or “no homogeneous” definition when a latent construct is introduced or investigated. In fact, many researchers report different definitions of a single concept and finally elaborate their own view and their own definition. To say it more provocatively, for some latent constructs, there are nearly as many definitions as there are researchers working on them. Hagger (2014) spotlighted the problem of the many overlapping constructs in psychological research and claimed that more guides to constructs are needed, as Skinner (1996) presented for constructs addressing issues of control. Mentioning the term-mingling problem, Skinner says that “when the same term is used to refer to different constructs, reviewers may conclude that findings are inconsistent or even contradictory, when in fact it is definitions that are inconsistent and contradictory” (Skinner, 1996, p. 550). Later, Skinner explicates this problem with the term “secondary control” (Skinner, 1996). In psychological science, the existence of different terms with an implicitly overlapping meaning and the existence of a single term with different meanings both entail difficulties for empirical research.

Due to a dominant focus on statistical methodology, psychologists tend to concentrate more on the inner consistency and congruency of latent constructs than on the valid description of psychological phenomena, as Michell (2013) already argued. Dealing with latent constructs, epistemology seems to be reduced to a halfhearted demand for generalizability, the demand for objectivity and simultaneously the ignoring of the researcher’s subjectivity and perspective, respectively. Theory, sometimes, seems to be reduced to considerations about the constructs that were measured in the study. This neglect leads to the problem of overlapping constructs, concepts and approaches in psychological research and makes it redundant and uncontrollably inexact. If objectivity and generalizability really would work with latent constructs, there would be no problem with overlap, redundancy and, last but not least, the replication crisis. So, should we say goodbye to latent constructs, objectivity or generalizability? Can we overcome the replication crisis taking into account epistemological considerations? Maybe we can solve some parts of it.

The argument is that for a capable, process-oriented and updatable validation of latent constructs that protects us against redundant concepts of psychological phenomena, we have to replace generalization, induction and deduction with a more natural and efficient approach to learning: assimilation and accommodation. In other words, the idea is to consider always that meeting statistical objectivity and generalizability of a construct does not mean that a theory is really true (Meehl, 1992). Statistically perfectly verified constructs also should be handled with theoretical and phenomenological reflection and perspective-taking. As soon as latent constructs depend on personal perspectives, objectivity and generalizability a strict induction-deduction-logic is excluded. However, latent constructs always depend on a perspective: In the best case, they depend on the perspective of an approach or a theory, but they are also influenced by the strategy to calculate them, by a single scientist or a group of scientists. In fact, we need a more honest approach to our latent constructs that explicate our automatized perspective-taking. With perspective-taking, I do not mean to let personal, subjective or political aspects influence a certain construct. It is meant, as a first step, to identify the potential influences that create the understanding of a construct. Not to identify the influences does not mean that the influences do not take place.

**FIGURE 1:**
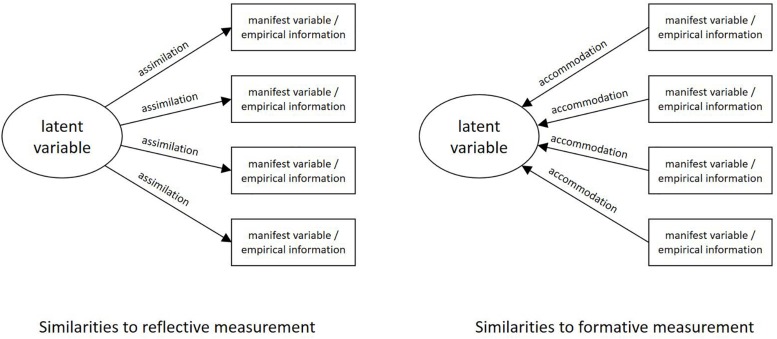
Similarities to reflective and formative measurements according to Edwards and Bagozzi (2000).

I argue that latent constructs should be handled flexibly like mental representations as Piaget (1976) and Baldwin (1906) before him proposed and investigated. Assimilation in the original psychological sense means that an outside object is adapted to an already existing mental representation. Accommodation, in contrast, means that a mental representation is adapted (newly created or actualized) to an object. In other words, assimilation means that a mental representation changes (via perception or action) an object, whereas accommodation means that an object changes a mental representation. Transferred to latent constructs, you can say that assimilating mental representations and latent constructs (e.g., via expertise or a measuring procedure) adapts psychological phenomena to the mental representation or the questionnaire’s concept. It is similar to Edwards and Bagozzi’s (2000) distinction between reflective and formative measurements of latent constructs. The authors describe reflective (assimilative) measures as something where “constructs are usually viewed as causes of measures” (Edwards and Bagozzi, 2000, p. 155), and further “[i]n some situations, measures are viewed as causes of constructs” (ibid). The latter they call formative (accommodative) measurement, which occurs especially when we “know” that the construct is not one-dimensional, such as socioeconomic status. Figure 1 shows the difference between the two approaches.

Assimilating here implies the ignoring of potential changes of phenomena, because latent constructs or mental representations cause the measurement. Accommodating latent constructs means that psychological phenomena are perceived less dependent from existing constructs or mental representations. Potential changes of psychological phenomena are not ignored, but they foster an actualization of the mental representation and, consequently, an actualization of the corresponding latent construct. It seems that this is one of the basics of good research practice that is applied anyhow.

## Our Problem With Induction, Deduction and Generalizability and Ellen Skinner’s Work

Why could it be an advantage to apply assimilation and accommodation instead of induction and deduction? In philosophy of science, induction means to infer generalized principles from specific empirical observations. Deduction means to infer the validity of specific empirical observations from a generalized principle. In contrast, the distinction of assimilation versus accommodation describes a different, very basic and adaptive strategy to generate knowledge. Induction and deduction describe logic-based inferring procedures, coming from a highly sophisticated epistemological tradition. However, assimilation and accommodation are closer to our natural knowledge-generating functions, or as Caligiore et al. (2014) would say, the processes have an intuitive power and occur automatically. What is most important here, in the context of assimilation and accommodation, generalizability does not have this absolute understanding of generalizability. Knowledge can formally depend on perspective, time, or place.

Applying the scientific induction concept, we falsely assume the generalized validity of mental representations without the possibility of testing them empirically. This was Popper’s main critique point of positivism and induction (Popper, 2005). However, even the deduction concept has a similar problem, because it assumes that a valid generalized mental representation already exists. This is the reason why Popper suggested to speak of tentative knowledge and not of secure scientific knowledge. Maybe the second problem is not so virulent for observable facts because exceptions are very obvious sooner or later. But it becomes difficult when we think, for example, about a mental representation of a personality trait which is non-observable but should be valid interculturally or over a time period of fifty or a hundred years. So, a proven theory fosters the assimilative mode. We apply “already existing” knowledge, also, for example, after a sophisticated inductive process. A too distinctive assimilative mode means that we tend to assimilate even if we should have received empirical hints to rethink – accommodate – our mental representations.

There is a further problem in developing latent constructs. I postulate that the development of latent constructs is based on very similar assimilative and accommodative processes such as children’s development of mental representations – for example, how a child develops his or her mental representation of a cat. He or she learns to say “cat” when he or she sees a cat. The child also says “cat” if he or she sees a dog or another animal that bears analogies to a cat, such as a marten. In this case, the child does not yet have a well-developed representation of a cat. In spite of this fact, the child tends to assimilate all objects which are more or less analogous to a cat. In order to fit the objects “dog” and “marten” to the scheme of a cat, micro-accommodative processes, as Piaget (1976) described them, are necessary to handle the discrepancies between the animals in an automatized process. Only when the child conducts non-automatic (and non-micro-) accommodative processes aimed at developing two new mental representations, one called “dog” and another called “marten,” is he or she able to distinguish the three objects “cat,” “dog,” and “marten” correctly. Using three schemes instead of one also implies that the automatically running micro-accommodative processes which accompany the perception of one of the three animals are no longer as extensive.

Skinner et al. (2003) show us how we can reduce micro-accommodative processes in psychological research. Investigating the different meanings of the term “coping,” the authors say: “[W]e focused on how these category systems were created. We considered about 100 schemes used during the past 20 years” (p. 218). The authors then make a lot of distinctions within the concept of coping. For example, they identified different functions of coping, topological distinctions as higher order categories of coping, effortful versus involuntary responses to stress, and so on. In sum, Skinner et al. (2003) reconsider the term “coping,” suggesting new understandings of the association of the different definitions, hierarchical connections and theoretical implications. As mentioned above, Skinner (1996) provided a similar “guide to constructs” for the term “control”: “The goal of this article is to collect control-related constructs and to organize them according to their definitions” (p. 550). In her article, Skinner differs between subjective and objective control, the experiences of control, motivations for control, agents, means and ends of control and means-ends, agent-means and agent-ends relations, respectively, and so forth. In fact, Skinner provides a theoretical integration of many independently developed constructs in order to enhance the validity of psychological research and to reduce replication problems due to definition fuzziness.

## Recent Concepts of Assimilation and Accommodation

Several approaches discuss assimilative and accommodative processes in different psychological contexts. The most basic one investigates them on a physiological information processing level. Fiedler (2001) and Fiedler et al. (2010) describe assimilation as a top-down process which is knowledge driven. In contrast, accommodation can be seen as a stimulus-driven bottom-up process.

The assimilative style in positive mood is by definition less contingent on large amounts of stimulus input than the accommodative style in negative mood. Conversely, assimilation includes the ability to enrich and elaborate a limited stimulus input through self-generated inferences, by going actively beyond the information given (Fiedler et al., 2010, p. 484).

These considerations go in line with further ideas, for example the so-called assimilation bias (Lord et al., 1979; Lord and Taylor, 2009). Lord and Taylor (2009) associate the assimilation bias with a tendency to over-generalize information that “allows people to develop assumptions and expectations even for specific objects that they have never encountered before” (p. 828). To some degree, the assimilation bias can be associated or even identified as an overlapping phenomenon with other biases, like the confirmation bias (Nickerson, 1998), which recently has been associated with the replication crisis (e.g., Lilienfeld, 2017). At the recent level of concretization, assimilation bias and confirmation bias reflect very similar phenomena: “As the term is used in this article and, I believe, generally by psychologists, confirmation bias connotes a less explicit, less consciously one-sided case-building process. It refers usually to unwitting selectivity in the acquisition and use of evidence” (Nickerson, 1998, p. 175). Analogical, “[b]iased assimilation occurs when perceptions of new evidence are interpreted in such a way as to be assimilated into preexisting assumptions and expectations” (Lord and Taylor, 2009, p. 827). Similar to both of these biases, the Einstellung effect, first investigated by Luchins (1942), plays a role when once found problem solutions are preferred to faster or easier solutions. Bilalić et al. (2010) showed that this effect takes place when experts are using their expertise (see also Bilalić, 2017), and researchers and scientists are assumed to be experts in using and applying theories and concepts.

Proulx and Heine (2010) discussed these phenomena on the level of philosophy and psychological research. They suppose the meaning maintenance model as an integrative framework, which focus on forced assimilative processes when a meaning making system is violated by external stimuli and argue this effect in the context of threat-compensation literature. In this context, “Assimilation is a common response to meaning threats because it’s fast and requires little in the way of cognitive resources” (Proulx and Heine, 2010, p. 894). Conversely, “accommodation is such a resource-heavy process, in the face of an anomaly people often do not have the wherewithal to begin to make any sense of what they’ve encountered” (ibid). For similar differences between assimilation and accommodation see Labouvie-Vief et al. (2010), where they focus on life-long-learning and the role of emotions:

Assimilation represents a low effort, automatic, and schematic processing mode, in which judgments are framed in a binary fashion of good or bad, right or wrong, and positive or negative. Regulation is oriented at dampening deviations from these binary evaluations. Accommodation, in contrast, involves a conscious and effortful unfolding, elaboration, and coordination of emotional schemas into complex knowledge structures (p. 87).

Looking at Piaget, how can it be that assimilation and accommodation are not always equilibrated but biased? Maybe because the two antagonists are not always balanced. Block (1982) was the first who proposed a different concept of equilibration that allows the idea of prolonged assimilative or accommodative forces, with appropriate consequences for our knowledge generation. In several later approaches, like in the assimilation bias and similar biases, assimilation and accommodation are not forced balanced (e.g., Hollon and Garber, 1988; Bosma and Kunnen, 2001; Fiedler, 2001).

## Block’s Approach of Equilibration, the Possibility to Over-Accommodate and Over-Assimilate and What We Can Learn From Neuro-Robotics

Piaget (1976) described equilibration as something that is reached automatically due to the subject’s adaptation to the world via assimilation and accommodation. Assimilation and accommodation, in their understanding, are balanced out and occur equally distributed. Coming from a personality-oriented perspective, Block (1982) suggested a different understanding of equilibration. Here, people differ in their way to approach equilibration with their environment: Whether a person tends to assimilate to reach equilibration with his or her environment, or he or she tends to accommodate to reach equilibration. Block associated people with prolonged assimilative efforts with the absence of the registration of discrepancies, with being too enthusiastic in the application of schemes, and with intolerance of ambiguity. In contrast, he characterizes people with prolonged accommodative efforts through their behavioral fluctuations, ever-changing perceptual-cognitive-action recognitions of possibilities, and intolerance of simplicity (Block, 1982, p. 292). Block’s suggestion to perceive assimilation, accommodation and equilibration differently is a helpful foundation to explain the phenomenon of over-assimilation or over-accommodation, which have been investigated in clinical research.

In clinical research, for example, a different understanding of equilibration is part of trauma research. Littleton and Grills-Taquechel (2011, p. 421), for example, describe the sub-optimal strategy of over-accommodation when dealing with traumas, which means a “maladaptive or extreme schema change” (see also Hollon and Garber, 1988; Krawczyk et al., 2017). Taking the definition of assimilation and accommodation by Aguilar and Pérez y Pérez (2015), over-accommodation clearly should be considered seriously as a relevant source of the replication crisis. They define assimilation processes in their neurorobotic system as “search of schemas in memory representing similar situations to the one described in the current-context.” (Aguilar and Pérez y Pérez, 2015, p. 31). Conversely, they see accommodation processes “as creation of new schemas and the modification of the existing ones as a result of dealing with unknown situations in the world” (Aguilar and Pérez y Pérez, 2015, p. 29).

Given that psychological phenomena are already described and investigated in literature, sometimes it would be better to read more before creating a new latent construct. Constructing new psychological constructs without a systematical scan of literature comes very close to a scientific over-accommodation, with many overlapping constructs and replication problems as a result. In contrast, if we transfer Block’s approach of equilibration to a fixed, generalized latent construct, which is measured by a questionnaire or test, the measurement is clearly associated with an assimilative mode and fosters the ignorance of discrepancies. Even if we can identify the quantity of measurement error, we do not know the quality of measurement error or unexplained variance. In many models, the amount of unexplained variance is higher than the amount of explained variance. Even effect sizes are no reliable identifier of the amount of measurement error (Loken and Gelman, 2017). Here, an accommodative process is needed, not only on a statistical level but also on conceptual, theoretical and epistemological levels.

To conclude, the development of latent constructs in a positivistic tradition via induction and deduction implies the demand of their generalizability. However, empirically, this is a status that hardly can be reached. Even more, it leads to many uncontrollably over-assimilated or over-accommodated and therefore overlapping latent constructs. Latent constructs are particularly affected by this problem because they are (1) non-observable, (2) only weakly dependent upon concrete behavior and therefore difficult to validate, (3) individually abstracted by us and therefore (4) more vulnerable to implicit subjectivity and assimilation and similar biases. I assume that the explication of assimilative and accommodative processes in empirical research methods helps to enhance the validity of latent constructs and to reduce the déjà-variable phenomenon as well as the poor replicability of our research; according to Block, “Assimilate if you can, accommodate if you must” (Block, 1982, p. 286). Following Block (1982), I summarized some causes and consequences of over-assimilation and over-accommodation when doing research with latent constructs (Table 1).

**TABLE 1:** Causes and consequences of over-assimilation and over-accommodation.

	**Over-assimilation**	**Over-accommodation**
Development and application of latent constructs	Ignorance of discrepancies and (e.g., societal or cultural) changes of constructs	Ignorance of already existing constructs

Problems concerning the validity of latent constructs	Implicit (unidentified) overlaps of constructs because one construct describes different phenomena	“Invention” of “new” constructs which describe already known phenomena without additional information (déjà-variables)

Scientists’ personal tendency	Intolerance of ambiguity	Intolerance of simplicity

Scientists’ needs	Need to defend their “own” construct	Need to develop their “own” construct

Speaking about these challenges seems to imply that many aspects of research practice need to be changed, but this is not the case. In fact, most field-tested research methods which are currently in use do a very good job: They are doubtless the most highly elaborated perspectives to refine and actualize constructs or theories. They allow the necessary professional distance to mental representations and therefore the potential to reduce over-assimilation or over-accommodation. However, research methods alone do not protect a researcher against over-assimilation and over-accommodation automatically. In the following, I set out some consequences and implications on the research method level, on the individual level and on the level of philosophy of science which could enhance the explication of well-balanced assimilative and accommodative research processes.

## Consequences at Research Method Level

The explication of assimilation and accommodation in research processes is accompanied by perspective-taking. Research methods should be seen as perspectives which provide a view on constructs or psychological phenomena. Sometimes, there seems to be a kind of confusion between research method and psychological phenomenon, especially when a phenomenon can be made evident by only one research method. This confusion of research methods with the construct itself plays a role in the emergence of overlapping constructs. Even more, as discussed above, scientific over-accommodation takes place when a new construct is introduced without scanning existing literature and the new construct can be measured by one research method. Measuring a construct as assimilative process again ignores discrepancies between the new construct, already existing constructs and the phenomenon itself. Given a validity study with a correlation of *r* = 0.7, still 51% of the variance remains unexplained, without any idea what this 51% could be (see Loken and Gelman, 2017). Many constructs overlap because they are each “found” by one research method, one style of thinking or one view of different disciplines. The real problem comes when the many overlapping constructs are not compared with each other on a theoretical level. Here, an exact differentiation between the construct, the view of the construct and the phenomenon is needed. Uher (2018) provides a highly elaborated guide which should be considered when measuring psychological phenomena.

## Consequences at the Level of Students and Scientists

One implication at the level of scientists is that they need well elaborated mental representations of theories and constructs to ensure a differentiation between theories, constructs and different views on a construct. The recent need to conduct reviews is a consequence of not having the same concepts in mind when talking about theories. Systematical reviews are one good solution to meet this growing problem in empirical research. For example, Morling and Evered (2006) brought some clarity into the research about secondary control. They reviewed 53 empirical articles which were published between 1985 and 2005, compared the definitions of secondary control which were used in the studies and proposed a definition of secondary control that should comprise all relevant aspects of the empirical work on the construct. Skinner (2007) could, based on Morling’s and Evered’s challenging but necessary work, provide even more theoretical clarity about the concept of secondary control. If Morling, Evered and Skinner had not done this work, many different perspectives on secondary control would still stand side by side and reinforce the problem of Hagger’s déjà variable. However, this is only one construct which was only used in a manageable area of research. Thus, the strategy is to be as well informed as possible about theories and constructs in psychology and neighboring disciplines. This implies an intensive theory-based education for students, which ensures a well elaborated development of mental representations of theories. Furthermore, students need trainings to develop the competence to consider and clearly discuss constructs, their interconnections and their connections to theories as well as the competence to identify their own relation to psychological phenomena. In order to foster the connection between the students’ mental representations and psychological phenomena, we must teach them the competence to distinguish between their own assimilative and accommodative modes. Additionally, well developed mental representations ensure a higher quality of their application in research practice, but also in clinical and other practices.

One of the most important but perhaps underestimated consequences for scientists is the point that responsibility for the validity of a construct or theory cannot be delegated to empirical research methods. They can help a scientist to accommodate his or her cognitive schemes when they facilitate a better or more specific view of a construct or theory. We have to take the consequences that Meehl (1992) already articulated: “No statistical procedure should be treated as a mechanical truth generator” (p. 152). The validity of a construct still depends on a scientist’s or a scientific community’s conclusions (see also Edelsbrunner and Dablander, 2018). The validity is maximized when they minimize over-accommodation or over-assimilation and other biases. One example comes from the psychotherapeutic research practice. There is a more than 20-year-old debate about how to integrate knowledge from different therapeutic schools. In this context, Wolfe (2001, 2008) suggested to develop this integration on both assimilative and accommodative integration, not only assimilative integration.

## Consequences for Philosophy of Science

All in all, there are not as many inconsistencies between positivistic thinking, critical rationalism, and systemic and constructivist epistemologies as often discussed. Rather, I assume, they describe different phases of a knowledge-generating process. Positivism describes the determination of a cognitive scheme on the basis of verification. It also justifies the assimilative-oriented process of induction or description of the world on the base of logico-mathematical principles, according to Block (1982, p. 286): “assimilate if you can.” Critical rationalism, besides preferring deduction, draws attention to the point that sometimes it is better to be in an accommodative mode in order to realize that a mental representation (theory, construct) does not necessarily fit the outside world: “accommodate if you must” (ibid). Systemic approaches show us the relevance of perspective and that our own perspective and our own behavior are part of and influencing those systems; or that some principles perhaps do not follow a logico-mathematical order. Ignoring it does not mean that it does not take place. Constructivism reminds us that, in fact, we cannot slip out of ego- and anthropocentrism, a point that should also not be ignored anymore. Thus, there is no need to take sides with any one of those ideas because all of them describe important aspects of the process of knowledge-generation. However, maybe we should take into account more the psychology of science (e.g., Gholson et al., 1989; Feist, 2006).

## Conclusion

In this paper, I want to show why I assume that replication problems in empirical psychology are not only due to statistical artifacts and methodological errors and why they are also caused by a lack of epistemological and theoretical clarity. I refer to validity problems that can arise from the use of latent constructs with a simultaneous positivist scientific orientation. As long as latent constructs are evaluated predominantly with regard to their calculation quality and too little with regard to their theoretical embeddedness in a coherent theory system, there is the potential that once calculated constructs are hardly falsified. Phenomena like Martin Hagger’s “déjà-variables” point to this problem.

I argue to meet this problem by taking a different epistemological perspective and propose why the use of assimilation and accommodation could be quite appropriate. Assimilation and accommodation are specific adaptation processes that describe and explain the development of cognitive and behavioral processes. They should therefore also be suitable for formalizing the further development of latent constructs. One important point is that there are already several examples from psychological research, such as Block’s personality approach or clinical work, where the concept of assimilation and accommodation helps to understand phenomena where over-assimilation and over-accommodation hinder the achievement of equilibrium and thus validity. The explicit integration of assimilation and accommodation in epistemology changes the perspective on theory development at the level of research methods, researchers and philosophy of science.

## Author Contributions

The author confirms being the sole contributor of this work and has approved it for publication.

## Conflict of Interest Statement

The author declares that the research was conducted in the absence of any commercial or financial relationships that could be construed as a potential conflict of interest.
